# Case Report: Combined transcatheter arterial embolization and aortic stent-graft have better efficacy for bronchial artery aneurysms

**DOI:** 10.3389/fcvm.2023.1328674

**Published:** 2024-01-08

**Authors:** Xiangui Li, Haoran Zhang, Wenqi Ma, Fanzhen Lv, Weimin Zhou

**Affiliations:** ^1^ Department of Vascular Surgery, The Second Affiliated Hospital, Jiangxi Medical College, Nanchang University, Nanchang, Jiangxi, China; ^2^ Queen Mary School, Jiangxi Medical College, Nanchang University, Nanchang, Jiangxi, China; ^3^ School of Ophthalmology and Optometry, Jiangxi Medical College, Nanchang University, Nanchang, China

**Keywords:** bronchial artery aneurysm, endovascular treatment, transcatheter arterial embolization, aortic stent-graft, cardiovascular surgery

## Abstract

Bronchial artery aneurysm (BAA) is a rare and fatal condition that requires immediate treatment. However, conventional surgical and transcatheter arterial embolization treatments are less effective. In the present case, a 76-year-old hypertensive woman was admitted with dizziness and diagnosed with an unruptured bronchial artery aneurysm, which was treated by transcatheter arterial embolization and aortic stent-graft. The patient's clinical status was stable during the 4-year follow-up. Simultaneously, we reviewed 79 research papers, analyzing past BAA cases for their etiology, symptoms, and treatment outcomes. We found that catheter arterial embolization and aortic stent-graft implantation, especially for BAA of short-necked and arterial tortuosity, demonstrate superior efficacy compared to other methods. Therefore, we consider this approach to be the preferred choice in clinical BAA treatment.

## 1 Introduction

Bronchial artery aneurysm (BAA) has a prevalence of less than 1% in patients examined by bronchial arteriography, but it can be fatal (1). Given the possible asymptomatic nature of BAA, timely intervention after diagnosis is imperative to mitigate the risk of rupture and subsequent massive hemoptysis (2, 3). As interventional techniques advance, transcatheter arterial embolization (TAE) is progressively replacing traditional surgery as the primary treatment method for BAA (4). However, TAE carries a risk of complications, including transverse myelitis, bronchial infarction, oesophageal-bronchial fistula, and even spinal cord ischemia (5). Furthermore, TAE poses the risk of inadequate bronchial artery embolization, increasing the probability of hemoptysis recurrence, particularly in cases involving persistent pulmonary tuberculosis (6). Consequently, in response to the limitations associated with TAE, the utilization of a combined strategy involving TAE and aortic stent-graft has emerged as a novel therapeutic option (7).

In this report, we successfully treated a 76-year-old female patient with a 25-mm diameter BAA using TAE and aortic stent-graft and the patient had a favorable prognosis without complications during a 4-year follow-up period. TAE and aortic stent-graft not only proved to be effective in the treatment of BAA in past reports but also demonstrated positive therapeutic effects and a good short-term prognosis in this case (8, 9).

In addition, the article reviews previous reports of BAA and provides a discussion of the etiology, symptoms, and treatment of a total of 85 patients with BAA, including the present case.

## 2 Case report

### 2.1 Case description

A 76-year-old woman presented to the neurology department with persistent dizziness and concerns regarding a possible brain lesion. The patient had a history of hypertension for over 20 years and a family history of cardiovascular disease. However, the patient claimed to have achieved long-term control of hypertension with amlodipine besylate tablets and had no personal or family history of psychiatric disorders or neurological diseases. Furthermore, a comprehensive physical examination was conducted on the patient. The results showed that the general appearance, head, neck, chest, abdomen, extremities, neurological system, and skin were all normal. However, the vital signs, namely, blood pressure and heart rate, were higher than normal at 175/62 mmHg and 103 beats per min, respectively. Therefore, due to the inability to rule out cardiovascular disease, vascular surgery staff participated in the consultation for this patient.

### 2.2 Diagnostic assessment

**Figure 1:**
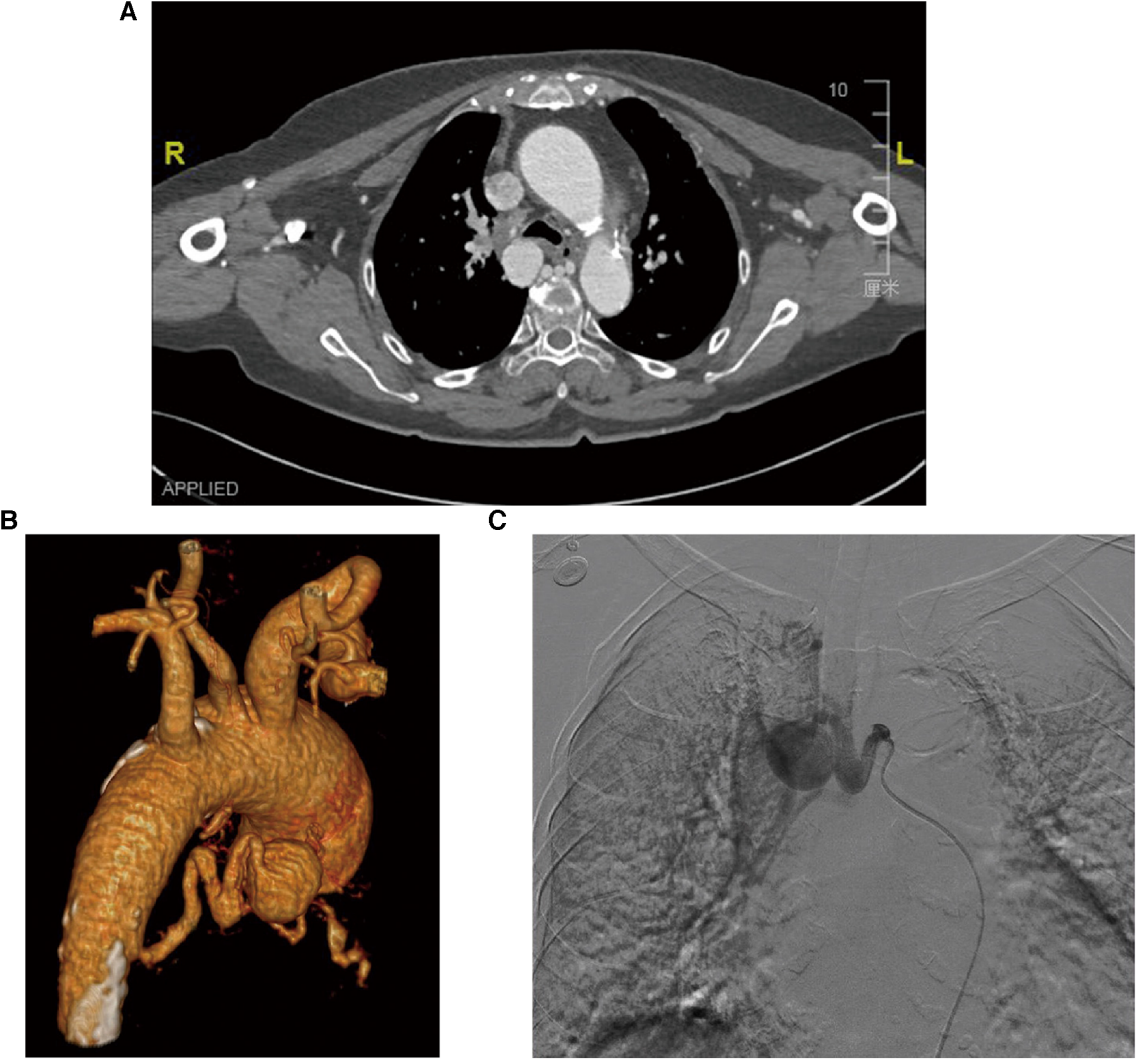
Diagnostic images of the patient with BAA. (**A**) The CT scan image of the chest shows an abnormally enhanced lesion in the mediastinum and dilated bronchial arteries near the descending aorta, but no entrapment or hemopneumothorax. (**B**) Three-dimensional reconstruction of the CT angiographic model shows a 25-mm-diameter BAA from the descending aorta with a dilated and tortuous inflow vessel. (**C**) Selective bronchial arteriography shows that the aneurysm was fed by a bronchial artery dilated by the descending aorta.

The suspicion of an aneurysm was first raised by an abnormal enhancement of a mediastinal lesion on a contrast-enhanced computed tomography (CT) scan of the chest (Figure 1A). The subsequent three-dimensional reconstruction of the CT angiogram showed a BAA with a 25-mm diameter and a dilated and tortuous inflow vessel from the right bronchial artery trunk (Figure 1B), which together with an aortic arch angiogram confirmed this diagnosis (Figure 1C).

### 2.3 Treatment

Following the puncture of the right femoral artery using the modified Seldinger technique, a 5 Fr arterial sheath and a 0.035-inch supersmooth guidewire were carefully inserted. Subsequently, aortography was conducted using a 5F pigtail catheter (Terumo, Tokyo, Japan) to assess the position of the opening of the aneurysmal inflow vessel of the bronchial artery. Super-selective angiography of the bronchial artery, performed with a 5F Cobra catheter (Terumo, Tokyo, Japan), confirmed the absence of spinal artery branch involvement and revealed a bronchial artery aneurysm with a diameter of approximately 25 mm.

**Figure 2:**
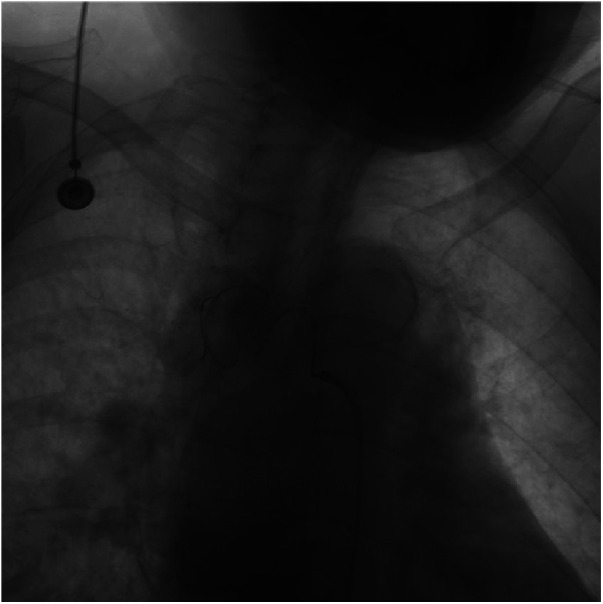
Angiogram during catheter intervention. Intraoperative angiography of the BAA shows that the catheter failed to enter the aneurysm due to tortuous arteries.

To achieve comprehensive embolization, a sandwich therapy plan was devised to target both the inflow and outflow vessels, as well as the aneurysm itself. However, due to the tortuosity of the inflow vessel, it was challenging to advance into the outflow vessel using a microcatheter (MC-PE28131, Terumo, Tokyo, Japan) and a 0.018-inch micro-guidewire (Figure 2). Consequently, a cautious and gradual injection of polyvinyl alcohol (PVA) foam embolic particles (500–700 μm, Cook Medical, Bloomington, USA) was performed at the aneurysm site until distal blood vessels were no longer visible in angiography. Subsequently, occlusion of the inflow vessel was achieved using multiple embolic coils (IMWCE-35-8-10/15, COOK Medical, Bloomington, USA). In a strategic move to prevent potential revascularization by collateral arteries from the thoracic aorta, an aortic stent-graft (Ankura^TM^ TAA Stent Graft System, TAA2626B080, Life Technology Sciences, Shenzhen, China) was deployed in the thoracic aorta segment at the BAA opening, with a 20% oversizing. Subsequent angiography demonstrated the disappearance of the BAA, confirming the successful occlusion of the aneurysm.

### 2.4 Follow-up

**Figure 3:**
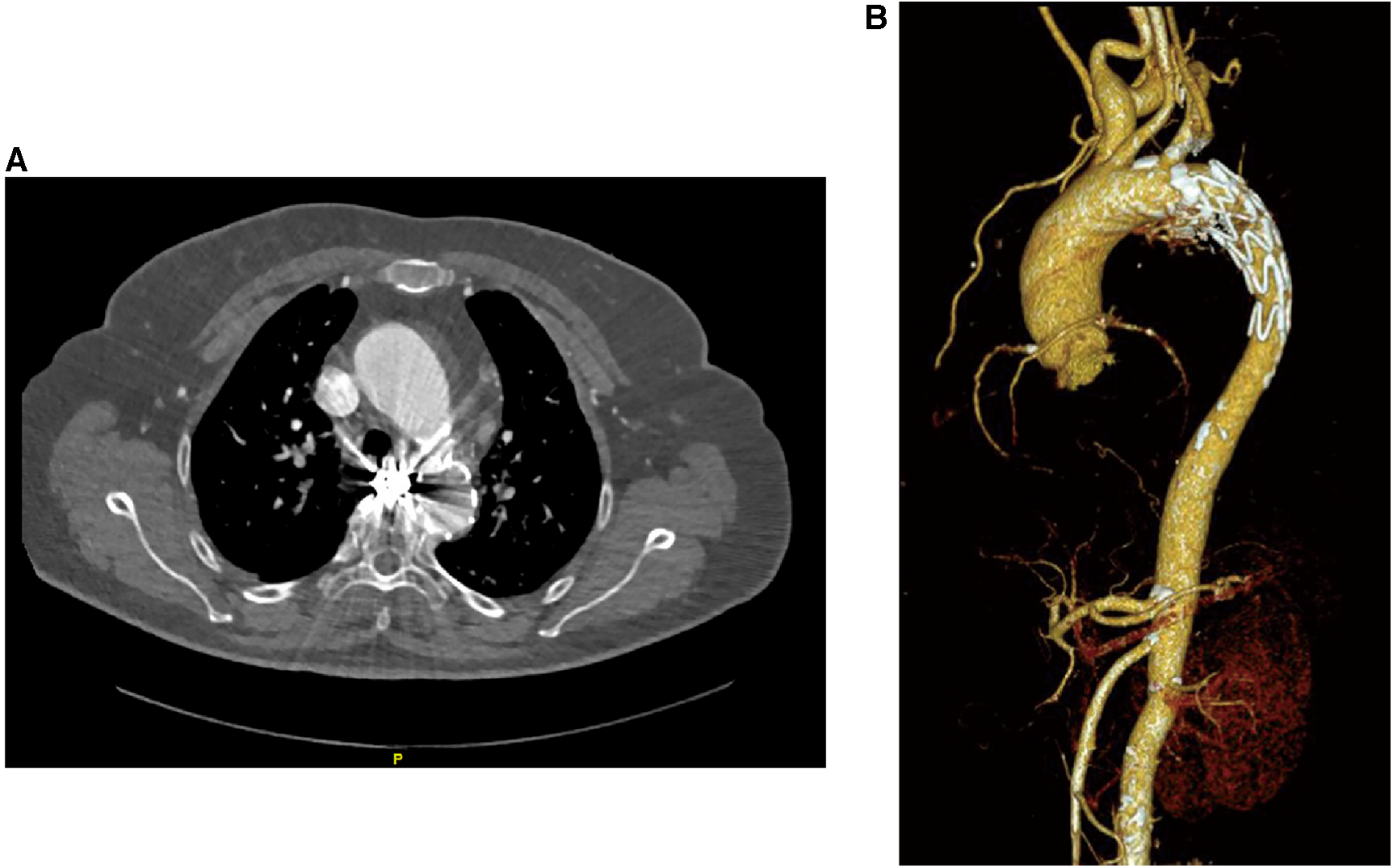
Patient's follow-up examination pictures after 1 year of treatment. (**A**) CT angiography was performed on the patient 1 year after treatment, and the scan suggests the presence of metallic artifacts from coils within the BAA. (**B**) Three-dimensional reconstruction of the vessel based on the CT angiography data shows no rupture of the BAA and no new collateral arteries supplying the BAA.

The patient was discharged from the hospital without any complications. Follow-up after 1 year, utilizing CT angiography and three-dimensional aorta reconstruction, revealed an absence of contrast filling in the BAA, indicating a positive prognosis during the 1-year follow-up (Figure 3). Despite the patient's reluctance to undergo further hospital-based examinations due to financial constraints, an ongoing assessment was sustained through telephone interviews conducted every year to evaluate the patient's clinical status until the fourth year. Throughout the series of telephone follow-ups, the patient consistently expressed satisfaction with the treatment outcomes and claimed no discomfort.

## 3 Timeline

**Figure 4:**
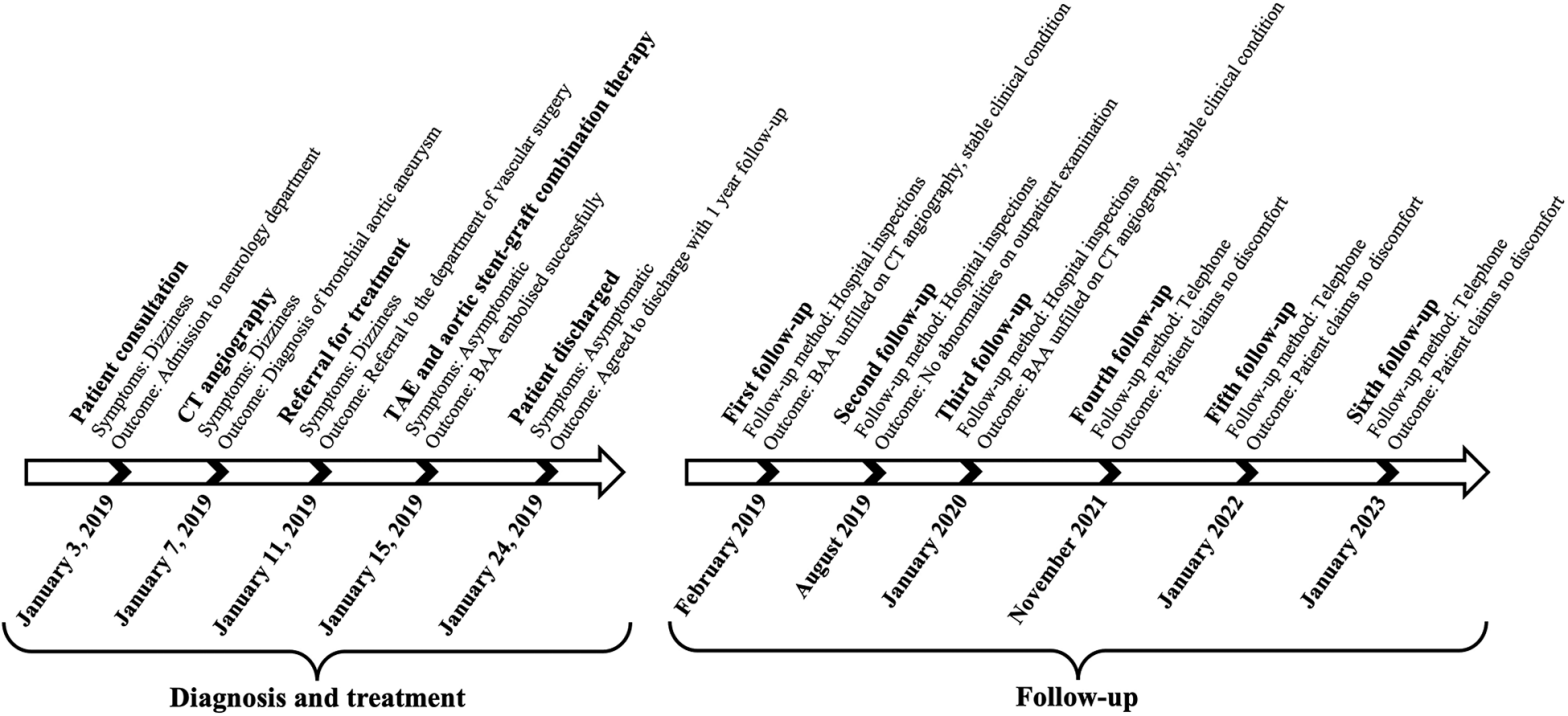
Timeline of treatment and follow-up.

The figure below illustrates the timeline for the diagnosis, treatment, and follow-up of this case (Figure 4).

## 4 Discussion

As a rare and primarily asymptomatic illness, BAA has an extremely high death rate if it ruptures and hence requires rapid treatment (1–3). In this case, a 76-year-old patient with hypertension presented with persistent headaches, prompting the performance of a contrast-enhanced CT examination that unveiled the presence of BAA with a diameter reaching 25 mm. Given the patient's advanced age, our therapeutic decision-making aimed to minimize potential complications. Consequently, a strategic choice was made to employ a less invasive intervention, opting for TAE over conventional surgical approaches (4). In the application of TAE, the sandwich therapy method emerges as the preferred approach due to its efficacy in averting bypass reflux by embolizing both inflow and outflow vessels (10). However, the catheter could not enter the outflow artery due to arterial tortuosity. Therefore, we employed a catheter to enter the BAA and induce embolization of the outflow artery by slowly injecting polyvinyl alcohol (PVA) particles to prevent blood reflux. Notably, coils are a safer option, but they present challenges when navigating through tortuous arteries. Concurrently, to mitigate the potential risks of coil dislocation and the washout of PVA particles by blood flow, we implemented an aortic stent-graft (8, 9). Notably, the application of an aortic stent-graft has demonstrated efficacy in isolating the thoracic aorta, preventing the formation of new collateral arteries and thereby impeding aneurysm refilling (9).

Furthermore, to obtain more clinical data and enhance our understanding of BAA, we searched for the keyword “bronchial artery aneurysm” using Pubmed and Google Scholar. Through the search, we selected a total of 85 cases from 79 articles for analysis (Supplementary Table 1). The sample consists of 41 women and 44 men, with a mean age of 57.35 years. The criteria for classifying the effectiveness and success of patient treatment were based on the Society of Interventional Radiology (SIR) guidelines (11).

The etiology of BAA is complex and yet not fully understood. However, current research suggests that its formation is caused by increased bronchial arterial blood flow and structural weakening of the vessel wall (8). According to reports, the etiology of BAA may be associated with hereditary factors, such as pulmonary sequestration and pulmonary artery agenesis. Additionally, acquired factors such as atherosclerosis, bronchiectasis, tuberculosis, and sepsis have been suggested as etiology (12). In the review, the most common cause for patients was bronchiectasis (19/85), followed by tuberculosis (7/85) and hypertension (6/85), which corroborates the findings of previous studies. Interestingly, the number of patients with mycotic aneurysm (5/85) was also significant in our review. However, mycotic aneurysms have rarely been noticed and reported before. By definition, the mycotic aneurysm is a dilatation of the arterial wall caused by bacterial, fungal, and viral infections (13). Pathogens tend to invade damaged arteries, leading to infection of the intima. Then, it leads to the rapid degradation of the deeper arteries, ultimately causing the formation of an aneurysm (14). Therefore, despite the complex and challenging etiology of BAA, patients with a history of cardiovascular disease, bronchiectasis, and tuberculosis are at an increased risk of developing BAA and require additional clinical attention. It is noteworthy that over the past two decades, the incidence of mycotic aneurysms as the etiology of BAA has been increasing each year. Thus, we should remain vigilant about this emerging trend in BAA etiology (13).

BAA not only has a complex etiology but also has a diverse clinical presentation (15). In our review, we observed that hemoptysis (28.24%, 24/85) was the most common clinical symptom in patients with BAA, followed by chest pain (18.82%, 16/85) and dysphagia (8.24%, 7/85). However, the main symptoms of BAA depend on its location (8). Intrapulmonary BAA commonly presents with hemoptysis, but there is a small proportion of asymptomatic cases. On the other hand, intrapulmonary BAA has been reported to cause dysphagia as the main symptom, although it is asymptomatic in most cases (16). It is worth noting that there are asymptomatic patients in all types of BAA, accounting for a certain percentage of patients in our review (14.12%, 12/85). These patients are usually detected incidentally on chest CT scans, so it is easy to delay treatment (17). Additionally, the presence or absence of BAA rupture can also affect the symptoms. Among patients with ruptured BAA, a significant proportion (40.00%, 10/25) experienced chest pain symptoms. In contrast, patients without ruptured BAA had a lower proportion of chest pain symptoms (6.98%, 6/86), with the most frequent symptom in these cases being haemoptysis, occurring in 17.44% (15/86) of patients. Notably, in the case review, the average diameter of BAA was 25.65 mm. Unruptured BAAs had an average diameter of 29.09 mm, while ruptured BAAs had a significantly smaller average diameter of 20.81 mm. The risk of BAA rupture appears to be inversely proportional to the diameter of the BAA. Therefore, if patients with BAA exhibit a smaller aneurysm diameter along with symptoms of chest pain, the likelihood of BAA rupture is significant. This necessitates urgent intervention for these patients.

So far, there are no standardized diagnostic and therapeutic guidelines for BAA, resulting in the use of a variety of diagnostic and therapeutic approaches (12). CT angiography stands out as the most frequently employed imaging modality for BAA diagnosis, with previous studies showing promise in MRI techniques (1, 18). TAE has emerged as the preferred treatment for BAA due to its safety, minimally invasive nature, and effectiveness (4). However, recent studies have demonstrated that combining aortic stent-graft with TAE yields better outcomes compared to TAE alone, particularly in patients with tortuous arteries and short necks (7). This combined approach not only mitigates the movement of TAE embolic coils but also effectively isolates the artery supplying the aneurysm, presenting a valuable advancement in BAA treatment strategies (19). In our case, the combination therapy yielded favorable outcomes and a positive short-term prognosis for BAA with a severely tortuous inflow artery, consistent with previous research. Furthermore, we conducted a comprehensive review of the success rates of different treatment methods. Among the 19 patients subjected to surgical intervention, 2 fatalities occurred, resulting in a mortality rate of 10.53%. In the cohort of 49 patients undergoing exclusive TAE, 9 experienced treatment failure, yielding a success rate of 81.63%. Notably, within the 13-patient group receiving combined therapy, the treatment success rate reached an impressive 100%. Therefore, combined therapy has a huge potential for the treatment of BAA. In contrast, certain studies suggest that the use of aortic stent-grafts may increase the risks of spinal cord ischemia and pseudoaneurysm rupture, warranting a cautious approach to their utilization (10, 20). However, due to the short follow-up period in this case, further monitoring is necessary to determine whether its long-term prognosis is favourable.

## 5 Conclusion

BAA has a low incidence but can be fatal. Based on the literature review and this case, we consider TAE and arterial stent implantation to be effective for BAA, particularly in cases with short necks and tortuous arteries. Given the complexity of BAA etiology and symptom diversity, our brief analysis of past literature provides insights into the causes and symptoms of BAA. This contributes to a better understanding of BAA and highlights the efficacy of TAE and aortic stent-graft.

## Data Availability

The original contributions presented in the study are included in the article/Supplementary Material, further inquiries can be directed to the corresponding authors.
