# Supplementary material for: Case Report: Combined transcatheter arterial embolization and aortic stent-graft have better efficacy for bronchial artery aneurysms
Source: Front Cardiovasc Med. 2024 Jan 8;10:1328674. doi: 10.3389/fcvm.2023.1328674 (PMC10801073; doi:10.3389/fcvm.2023.1328674)
Supplement: Supplementary file 1 [file Table1.docx]

Table 1 Detailed data of 85 case reports of Bronchial artery aneurysms.

| Author | Year | Age | Sex | Etiology | Size (mm) | Symptoms | Rupture | Treatment | Outcome | Follow up Duration |
| --- | --- | --- | --- | --- | --- | --- | --- | --- | --- | --- |
| Hall et al(1) | 1977 | 56 | M | Bronchiectasis | – | Shock | Yes | Surgery | Successful | – |
| Chantepie et al(2) | 1980 | 15 days | F | Mycotic aneurysm aneurysm | 7 | Hemoptysis | No | Surgery | Died | – |
| Abet et al(3) | 1981 | 50 | M | Pulmonary sequestration | – | Chest pain | Yes | Surgery | Died | – |
| Osada et al(4) | 1986 | 62 | F | Bronchiectasis | 30 | Hemoptysis | No | Surgery | Successful | – |
| Taguchi et al(5) | 1987 | 3 | M | Arteriovenous malformations | – | Hemoptysis | No | TAE | Failed | 5 years |
| Remy–Jardin et al(6) | 1991 | 50 | F | Bronchiectasis | 10 | Hemoptysis | Yes | TAE | Failed | 4 years |
| Connolly et al(7) | 1994 | 2 | F | Pseudoaneurysm (hyperimmunoglobulinemia E) | – | Hemoptysis | – | TAE | Successful | – |
| Komoda et al(8) | 1994 | 57 | F | – | 15 | Asymptomatic | – | TAE | Successful | 2 years |
| Ishizaki et al(9) | 1995 | 22 | F | Systemic vascular abnormality | 5 | Back pain | Yes | TAE | Successful | – |
| Ishizaki et al(9) | 1995 | 25 | F | Vascular malformations | 50 | Hemothorax | Yes | TAE | Successful | – |
| Hoffman(10) | 1996 | 70 | M | Atherosclerosis | – | Chest pain | Yes | TAE | Failed | – |
| Siegler et al(11) | 1996 | 60 | M | Mycotic aneurysm | 7 | Hemoptysis | – | TAE | Failed | – |
| Kalangos et al(12) | 1997 | 50 | M | Degenerative | 15 | Aortic dissection | Yes | Surgery | Successful | – |
| Oka et al(13) | 1997 | 54 | F | – | 10 | Tumor | – | TAE | Successful | 1 month |
| Sakai et al(14) | 1998 | 72 | M | Bronchiectasis | 25 | Asymptomatic | No | TAE+SG | Successful | 7 months |
| Yanagihara et al(15) | 1998 | 64 | F | Bronchiectasis | 22 | Hemoptysis | – | TAE | Successful | 6 years |
| Sancho et al(16) | 1999 | 50 | M | Agenesis | 40 | Chest pain | No | TAE | Failed | 6 months |
| Yanagihara et al(15) | 1999 | 64 | F | Bronchiectasis | 22*14 | Hemoptysis | No | TAE | Failed | 6 years |
| Yanagihara et al(15) | 1999 | 64 | F | Bronchiectasis | 30 | Hemoptysis | No | TAE | Failed | 3 years |
| Vernhet et al(17) | 1999 | 57 | F | Bronchiectasis | – | Hemoptysis | No | TAE | Successful | 2 years |
| Braks et al(18) | 1999 | 41 | F | Bronchiectasis | – | – | – | TAE | Successful | – |
| Shimokawa et al(19) | 2000 | 25 | F | – | – | Back pain, shock | – | TAE | Successful | 7 years |
| Pugnale et al(20) | 2001 | 72 | M | COPD | 15 | Epigastric pain | Yes | TAE | Successful | 1 month |
| Sakuma et al(21) | 2001 | 63 | F | – | 30 | Asymptomatic | – | TAE | Successful | 18 months |
| Tringali et al(22) | 2002 | 48 | F | – | 45*35*15 | Dysphagia | No | Surgery | Successful | 6 months |
| Kasashima et al(23) | 2003 | 79 | M | Pulmonary tuberculosis | 60 | Hoarseness | No | SG | Successful | 18 months |
| Fukunaga et al(24) | 2003 | 60 | M | – | 40 | Chest pain | No | TAE | Successful | 4 years |
| Suen et al(25) | 2003 | 50 | F | Sarcoidosis | – | Hemothorax | Yes | Surgery | Successful | 6 months |
| Tanaka et al(26) | 2003 | 78 | M | – | 40 | Tumor | – | Surgery | Successful | – |
| Chatterjee et al(27) | 2004 | 59 | M | – | – | Chest pain, shock | Yes | TAE | Successful | 8 days |
| Karmy–Jones et al(28) | 2005 | 78 | F | Tuberculosis | 16 | Hemothorax | Yes | TAE | Successful | 11 months |
| Kalva et al(29) | 2006 | 29 | M | Bronchiectasis | 10 | Hemoptysis | No | TAE | Successful | 2 years |
| Kalva et al(29) | 2006 | 36 | M | Bronchiectasis | 13 | Cough | No | TAE | Successful | 5 months |
| Aburano et al(30) | 2006 | 30 | F | – | 20 | Asymptomatic | No | TAE | Successful | 3 months |
| Tsolaki et al(31) | 2007 | 76 | F | Bronchiectasis | 40 | Asymptomatic | No | TAE+Surgery | Successful | 4 months |
| Sanchez et al(32) | 2007 | 69 | F | Pulmonary tuberculosis | 40 | Dysphagia | No | TAE+SG | Successful | 3 days |
| Lin et al(33) | 2008 | 37 | M | – | 40*40 | Chest pain | No | Surgery | Successful | 6 weeks |
| Cecka et al(34) | 2008 | 50 | M | – | 10 | Epigastric pain | – | TAE | Successful | 2 years |
| Lioulias et al(35) | 2008 | 22 | F | Bronchotomy, mycotic aneurysm | 3 | Hemoptysis | – | Surgery | Successful | 4 months |
| Yajima et al(36) | 2008 | 69 | F | – | 70 | Cough | – | TAE | Failed | 2 years |
| Mizuguchi et al(37) | 2009 | 77 | F | Bronchiectasis | 30 | Shock | Yes | TAE | Successful | 9 months |
| Takahashi et al(38) | 2010 | 74 | M | Bronchiectasis | 60 | Asymptomatic | No | TAE+SG | Successful | 6 months |
| Misselt et al(39) | 2010 | 53 | M | Non–Hodgkin lymphoma | 12 | Asymptomatic | No | TAE | Successful | 3 months |
| Hu et al(40) | 2011 | 67 | M | Hypertension | 40*30 | Chest pain | Yes | TAE+SG | Successful | 1 year |
| Bouayad et al(41) | 2011 | 80 | M | Pulmonary tuberculosis | 46 | Dysphagia | No | TAE+SG | Successful | 16 months |
| Seo et al(42) | 2011 | 41 | M | Pulmonary tuberculosis | – | Chest pain | Yes | TAE | Successful | 15 days |
| Karmakar et al(43) | 2011 | 56 | M | Tuberculosis | 5 | Hemoptysis | – | TAE | Successful | 2 months |
| Tsuboi et al(44) | 2011 | 82 | M | Bronchiectasis | 33 | Hemoptysis | No | TAE | Successful | 11 days |
| De Dominicis et al(45) | 2011 | 44 | F | Right pulmonary agenesis | 6 | Chest pain | No | TAE+Surgery | Successful | 6 months |
| Guzzardi et al(46) | 2012 | 66 | M | Bronchiectasis | 40 | Dysphagia | No | TAE+SG | Successful | 6 months |
| Hori et al(47) | 2012 | 78 | M | – | 10 | Chest pain | Yes | TAE | Successful | 1 week |
| Tan et al(48) | 2012 | 53 | M | – | 42 | Cough | No | Surgery | Successful | – |
| Kim et al(49) | 2013 | 79 | F | – | 7 | Chest pain | Yes | TAE+Surgery | Successful | 1 year |
| Martinez et al(50) | 2013 | 70 | M | – | 15 | Hemomediastinum | Yes | TAE | Successful | – |
| Vosse et al(51) | 2014 | 76 | F | Acute rheumatic fever | 55 | Chest pain | Yes | TAE | Successful | 6 weeks |
| Kim et al(52) | 2014 | 73 | F | Bronchiectasis | 32 | Hemoptysis | No | TAE+SG | Successful | 2 years |
| Cheng et al(53) | 2014 | 59 | F | Cirrhosis | 4 | Cough | No | – | Failed | – |
| Nakamura et al(54) | 2014 | 63 | M | – | 25 | Tumor | No | Surgery | Successful | 6 months |
| Xiaowei et al(55) | 2014 | 44 | F | – | – | Hemoptysis | Yes | Surgery | Successful | – |
| Kim et al(56) | 2015 | 55 | M | Hypertension | 10 | Chest pain | Yes | TAE | Successful | 1 year |
| Rognoni et al(57) | 2015 | 58 | M | Hypertension, COPD | 35*44 | Asymptomatic | No | TAE | Successful | – |
| Samura et al(58) | 2015 | 77 | M | – | 53 | Asymptomatic | No | TAE+SG | Successful | 2 years |
| Shi et al(59) | 2015 | 52 | M | – | 25 | Chest pain | Yes | TAE | Successful | 1 year |
| Divisi et al(60) | 2015 | 75 | M | – | 6 | Hemothorax | Yes | No | Successful | 2 years |
| Kitami et al(61) | 2015 | 63 | F | – | – | Tumor | No | Surgery | Successful | 35 days |
| Mahmood et al(62) | 2015 | 73 | M | – | 20 | Hemoptysis | No | TAE | Successful | 1 year |
| Di et al(63) | 2016 | 50 | F | Hypertension | 25*22 | Chest pain | No | TAE+SG | Successful | 4 months |
| Izaaryene et al(64) | 2016 | 59 | F | Uterine carcinoma | 55 | Dysphagia | No | TAE | Successful | 2 years |
| Latip et al(65) | 2016 | 61 | F | Mycotic aneurysm | – | Hemoptysis | No | Surgery | Successful | – |
| Fiorucci et al(66) | 2016 | 89 | M | – | 38 | Asymptomatic | No | SG | Successful | 6 months |
| Sato et al(67) | 2016 | 59 | M | Tracheal stent migration | – | Hemoptysis | Yes | TAE | Failed | – |
| Song et al(68) | 2016 | 59 | M | – | 18 | Chest pain, Dysphagia | No | TAE+SG | Successful | 3 months |
| Wang et al(69) | 2016 | 66 | M | – | – | Hemomediastinum | Yes | TAE | Successful | – |
| Salamone et al(70) | 2017 | 63 | M | COPD | 9 | Hemoptysis | No | TAE | Successful | 2 years |
| Matsumoto et al(71) | 2017 | 72 | F | Malignant lymphoma, Heumatoid arthritis | 30 | Asymptomatic | No | SG | Successful | 2 years |
| Hsieh et al(72) | 2017 | 63 | F | – | 12 | Dyspnea | No | TAE | Successful | 1 year |
| Salamone et al(70) | 2017 | 63 | M | COPD | 9 | Hemoptysis | No | TAE | Successful | 2 years |
| Ueda et al(73) | 2017 | 77 | F | Mycotic aneurysm | 3 | Hemoptysis | – | TAE | Successful | 7 months |
| Wang et al(74) | 2018 | 23 | M | – | 28 | Hemoptysis | No | TAE+SG | Successful | 4 months |
| Goh et al(75) | 2019 | 58 | M | Hypertension | 30*32*29 | Hemoptysis | No | TAE | Successful | 3 months |
| Li et al(76) | 2019 | 56 | F | Interstitial lung disease, Pulmonary tuberculosis | 40 | Dyspnea | No | TAE | Successful | 6 months |
| Present case | 2019 | 76 | F | Hypertension | 25 | Asymptomatic | No | TAE+SG | Successful | 4 years |
| Chen Y et al(77) | 2021 | 46 | F | Bronchiectasis | 15 | Bronchiectasis | No | TAE+SG | Successful | 1 month |
| Balar AB et al(78) | 2022 | 72 | F | – | 4.2*5.5*6.7 | Dysphagia, fever | No | TAE | Successful | – |
| Cho et al(79) | 2023 | 72 | M | Pneumonia | 24 | Dizziness, dysarthria | Yes | TAE | Successful | – |

*SG: Stent Graft; COPD: Chronic Obstructive Pulmonary Disease; “–”: Data not available*

References

1. Hall RJ, Miller GA, Kerr IH. Ruptured Bronchial Artery Aneurysm Mimicking Aortic Dissection. *Br Heart J* (1977) 39(8):909-10. doi: 10.1136/hrt.39.8.909.

2. Chantepie A, Robert M, Pelletier J, Gold F, Mercier C, Lacombe A, et al. [Mycotic Aneurysm of Bronchial Artery. Apropos of a Case in an Infant]. *Chir Pediatr* (1980) 21(6):407-10.

3. Abet D, Pietri J. [Ruptured Bronchial Artery Aneurysm Simulating Dissection of the Aorta in a Patient with Bronchopulmonary Sequestration (Author's Transl)]. *J Chir (Paris)* (1981) 118(12):743-6.

4. Osada H, Kawada T, Ashida H, Sodemoto Y, Noguchi T. Bronchial Artery Aneurysm. *The Annals of thoracic surgery* (1986) 41(4):440-2.

5. Taguchi T, Ikeda K, Kume K, Nagasaki A, Goto S, Matsuo S, et al. Isolated Unilateral Absence of Left Pulmonary Artery with Peribronchial Arteriovenous Malformation Showing Recurrent Hemoptysis. *Pediatr Radiol* (1987) 17(4):316-8. doi: 10.1007/bf02388246.

6. Remy-Jardin M, Remy J, Ramon P, Fellous G. Mediastinal Bronchial Artery Aneurysm: Dynamic Computed Tomography Appearance. *Cardiovasc Intervent Radiol* (1991) 14(2):118-20. doi: 10.1007/bf02577709.

7. Connolly B, Manson D, Khattak S, Burrows P. Bronchial Artery Aneurysm in Hyperimmunoglobulinemia E Syndrome. *Pediatr Radiol* (1994) 24(8):592-3. doi: 10.1007/bf02012742.

8. Komoda K, Hujii Y, Nakajima T, Abe K, Hamada Y, Niitu K, et al. A Ruptured Thymic Branch Aneurysm Mimicking a Ruptured Aortic Aneurysm, with Associated Bronchial Artery Aneurysms: Report of a Case. *Surg Today* (1994) 24(3):258-62. doi: 10.1007/bf02032898.

9. Ishizaki N, Shimokawa S, Tanaka K, Taira A, Onohara S, Tabata M, et al. Ruptured Bronchial Artery Aneurysm Associated with Pleural Telangiectasis and Tortuous Portal Obstruction: Report of a Case. *Surg Today* (1995) 25(9):852-4. doi: 10.1007/bf00311467.

10. Hoffmann V, Ysebaert D, De Schepper A, Colpaert C, Jorens P. Acute Superior Vena Cava Obstruction after Rupture of a Bronchial Artery Aneurysm. *Chest* (1996) 110(5):1356-8. doi: 10.1378/chest.110.5.1356.

11. Siegler CM, Mason AC, Clouston JC. Bronchial Artery Aneurysms in Association with Mycobacterium Avium Intracellulare Complex. *Journal of thoracic imaging* (1996) 11(2):155-7.

12. Kalangos A, Khatchatourian G, Panos A, Faidutti B. Ruptured Mediastinal Bronchial Artery Aneurysm: A Dilemma of Diagnosis and Therapeutic Approach. *J Thorac Cardiovasc Surg* (1997) 114(5):853-6. doi: 10.1016/s0022-5223(97)70094-1.

13. Oka M, Fukuda M, Terashi K, Takatani H, Narasaki F, Nakano R, et al. Bronchial Artery Aneurysm as a Cause of Atelectasis. *Intern Med* (1997) 36(12):917-9. doi: 10.2169/internalmedicine.36.917.

14. Sakai T, Razavi MK, Semba CP, Kee ST, Sze DY, Dake MD. Percutaneous Treatment of Bronchial Artery Aneurysm with Use of Transcatheter Coil Embolization and Thoracic Aortic Stent-Graft Placement. *J Vasc Interv Radiol* (1998) 9(6):1025-8. doi: 10.1016/s1051-0443(98)70445-2.

15. Yanagihara K, Ueno Y, Kobayashi T, Isobe J, Itoh M. Bronchial Artery Aneurysm. *Ann Thorac Surg* (1999) 67(3):854-5. doi: 10.1016/s0003-4975(98)01347-2.

16. Sancho C, Dominguez J, Escalante E, Hernandez E, Cairols M, Martinez X. Embolization of an Anomalous Bronchial Artery Aneurysm in a Patient with Agenesis of the Left Pulmonary Artery. *Journal of Vascular and Interventional Radiology* (1999) 10(8):1122-6. doi: 10.1016/s1051-0443(99)70202-2.

17. Vernhet H, Bousquet C, Jean B, Lesnik A, Durand G, Giron J, et al. Bronchial Aneurysms Mimicking Aortic Aneurysms: Endovascular Treatment in Two Patients. *Cardiovasc Intervent Radiol* (1999) 22(3):254-7. doi: 10.1007/s002709900378.

18. Braks E, Pauleit D, Strunk H, Schild H. [Diagnosis and Therapy of Bronchial Artery Aneurysm]. *Rofo* (1999) 170(1):123-4. doi: 10.1055/s-2007-1011020.

19. Shimokawa S, Ishizaki N, Watanabe S-i. Ruptured Bronchial Artery Aneurysm. *The Annals of Thoracic Surgery* (2000) 69(5):1641. doi: 10.1016/S0003-4975(99)01517-9.

20. Pugnale M, Portier F, Lamarre A, Halkic N, Riis HB, Wicky S, et al. Hemomediastinum Caused by Rupture of a Bronchial Artery Aneurysm: Successful Treatment by Embolization with N-Butyl-2-Cyanoacrylate. *J Vasc Interv Radiol* (2001) 12(11):1351-2. doi: 10.1016/s1051-0443(07)61563-2.

21. Sakuma K, Takase K, Saito H, Zuguchi M, Tabayashi K. Bronchial Artery Aneurysm Treated with Percutaneous Transluminal Coil Embolization. *Jpn J Thorac Cardiovasc Surg* (2001) 49(5):330-2. doi: 10.1007/bf02913144.

22. Tringali S, Tiffet O, Berger JL, Cuilleret J. Bronchial Artery Aneurysm Disguised as a Leiomyoma of the Esophagus. *Ann Thorac Surg* (2002) 73(2):632-3. doi: 10.1016/s0003-4975(01)03121-6.

23. Kasashima F, Endo M, Kosugi I, Matsumoto Y, Abe Y, Sasaki H, et al. Mediastinal Bronchial Artery Aneurysm Treated with a Stent-Graft. *J Endovasc Ther* (2003) 10(2):381-5. doi: 10.1177/152660280301000235.

24. Fukunaga A, Okushiba S, Ohno K, Kitashiro S, Kawarada Y, Shitinohe T, et al. Mediastinal Bronchial Artery Aneurysm with Hematemesis. *Dis Esophagus* (2003) 16(4):328-31. doi: 10.1111/j.1442-2050.2003.00360.x.

25. Suen HC, Dumontier CC, Boeren J, Charland W, Daily BB. Ruptured Bronchial Artery Aneurysm Associated with Sarcoidosis. *J Thorac Cardiovasc Surg* (2003) 125(5):1153-4. doi: 10.1067/mtc.2003.196.

26. Tanaka K, Ihaya A, Horiuci T, Morioka K, Kimura T, Uesaka T, et al. Giant Mediastinal Bronchial Artery Aneurysm Mimicking Benign Esophageal Tumor: A Case Report and Review of 26 Cases from Literature. *J Vasc Surg* (2003) 38(5):1125-9. doi: 10.1016/s0741-5214(03)00707-9.

27. Chatterjee A, Ghosh S, Salhiyyah K, Gaines P, Rocco G. A Rare Presentation of a Ruptured Bronchial Artery Aneurysm. *Thorax* (2004) 59(10):912. doi: 10.1136/thx.2004.028589.

28. Karmy-Jones R, Hastreiter D, Burdick T. Hemothorax Complicating Bronchial Artery Aneurysm. *Can Respir J* (2005) 12(5):279-81. doi: 10.1155/2005/953023.

29. Kalva SP, Wicky S. Mediastinal Bronchial Artery Aneurysms: Endovascular Therapy in Two Patients. *Catheter Cardiovasc Interv* (2006) 68(6):858-61. doi: 10.1002/ccd.20902.

30. Aburano H, Kawamori Y, Horiti Y, Kitagawa K, Sanada J, Matsui O. Bronchial Artery Aneurysm Embolization with Nbca. *Cardiovasc Intervent Radiol* (2006) 29(6):1141-3. doi: 10.1007/s00270-005-0166-1.

31. Tsolaki E, Salviato E, Coen M, Galeotti R, Mascoli F. Double Right Bronchial Artery Aneurysm Treated with Combined Procedures. *Eur J Vasc Endovasc Surg* (2007) 34(5):537-9. Epub 20070716. doi: 10.1016/j.ejvs.2007.05.011.

32. Sanchez E, Alados P, Zurera L, Canis M, Muñoz I, Casares J, et al. Bronchial Artery Aneurysm Treated with Aortic Stent Graft and Fibrin Sealant. *Ann Thorac Surg* (2007) 83(2):693-5. doi: 10.1016/j.athoracsur.2006.06.082.

33. Lin J, Wood DE. Bronchial Artery Aneurysm Refractory to Transcatheter Embolization. *Ann Thorac Surg* (2008) 86(1):306-8. doi: 10.1016/j.athoracsur.2008.01.033.

34. Cecka F, Raupach J, Hoffmann P, Motycka P, Stetina M, Neoral C. Ruptured Bronchial Artery Aneurysm: Case Report. *Acta Medica (Hradec Kralove)* (2008) 51(4):241-3.

35. Lioulias A, Misthos P, Kokotsakis J, Papagiannakis G, Skouteli E. Recurrent Massive Hemoptysis Due to Postbronchotomy Bronchial Artery Aneurysm: A Case Report. *Can Respir J* (2008) 15(3):127-8. doi: 10.1155/2008/712302.

36. Yajima N, Tsutsui H, Yoshioka T, Kasai H, Tomita T, Kumazaki S, et al. Gigantic Bronchial Artery Aneurysm Treated with Transcatheter Arterial Embolization: A Case Report. *Angiology* (2008) 59(6):757-60. Epub 20080402. doi: 10.1177/0003319707306150.

37. Mizuguchi S, Inoue K, Kida A, Isota M, Hige K, Aoyama T, et al. Ruptured Bronchial Artery Aneurysm Associated with Bronchiectasis: A Case Report. *Ann Thorac Cardiovasc Surg* (2009) 15(2):115-8.

38. Takahashi Y, Tsutsumi Y, Monta O, Kohshi K, Ohashi H, Shimamura K, et al. Stent Grafting for Giant Bronchial Artery Aneurysm Disguised as an Aneurysm with Multiple Feeding Arteries. *Ann Thorac Surg* (2010) 89(5):1652-4. doi: 10.1016/j.athoracsur.2009.10.003.

39. Misselt AJ, Krowka MJ, Misra S. Successful Coil Embolization of Mediastinal Bronchial Artery Aneurysm. *J Vasc Interv Radiol* (2010) 21(2):295-6. doi: 10.1016/j.jvir.2009.10.030.

40. Hu CX, Huang S, Xu ZW, Chen W, Huang JS, Fu Z. Combination of Aortic Stent-Graft and Arterial Embolization for Ruptured Bronchial Artery Aneurysm. *Ann Thorac Surg* (2011) 92(2):e19-21. Epub 2011/08/02. doi: 10.1016/j.athoracsur.2011.02.082.

41. Bouayad M, Bagan P, Brian E, Benabdesselam A, Couffinhal JC. [Mediastinal Bronchial Artery Aneurysm: Treatment with Stent-Graft]. *J Mal Vasc* (2011) 36(1):45-9. Epub 20101204. doi: 10.1016/j.jmv.2010.10.003.

42. Seo YH, Kwak JY. Spontaneous Hemomediastinum and Hemothorax Caused by a Ruptured Bronchial Artery Aneurysm. *Korean J Thorac Cardiovasc Surg* (2011) 44(4):314-7. Epub 20110818. doi: 10.5090/kjtcs.2011.44.4.314.

43. Karmakar S, Nath A, Neyaz Z, Lal H, Phadke RV. Bronchial Artery Aneurysm Due to Pulmonary Tuberculosis: Detection with Multidetector Computed Tomographic Angiography. *J Clin Imaging Sci* (2011) 1:26. Epub 20110519. doi: 10.4103/2156-7514.81293.

44. Tsuboi R, Oki M, Saka H. Mediastinal Bronchial Artery Aneurysm Mimicking a Subcarinal Tumor. *Respiration* (2011) 82(2):207-8. Epub 20110531. doi: 10.1159/000327720.

45. De Dominicis F, Leborgne L, Raymond A, Berna P. Right Pulmonary Artery Agenesis and Coronary-to-Bronchial Artery Aneurysm. *Interact Cardiovasc Thorac Surg* (2011) 12(3):507-9. Epub 20101220. doi: 10.1510/icvts.2010.250795.

46. Guzzardi G, Cerini P, Fossaceca R, Commodo M, Micalizzi E, Carriero A. Endovascular Treatment of Bronchial Artery Aneurysm with Aortic Stent-Graft Placement and Coil Embolization. *Ann Vasc Surg* (2012) 26(7):1013.e5-8. doi: 10.1016/j.avsg.2012.02.021.

47. Hori D, Noguchi K, Nomura Y, Tanaka H. Successful Endovascular Treatment of Ruptured Bronchial Artery Aneurysm. *Asian Cardiovascular and Thoracic Annals* (2013) 21(5):615-7.

48. Tan AK, Thirugnanam A. Giant Bronchial Artery Aneurysm Presenting as a Right Hilar Mass. *Asian Cardiovascular and Thoracic Annals* (2013) 21(2):227-30.

49. Kim HJ, Son HS, Cho SB, Kim KT. Development of a Life-Threatening Mediastinal Hematoma from A ruptured Bronchial Artery Aneurysm. *J Thorac Cardiovasc Surg* (2013) 146(5):e47-9. Epub 20130909. doi: 10.1016/j.jtcvs.2013.07.034.

50. Martínez E, Villar I, Pérez S, León M, Rubio T, Garcia C, editors. Hemomediastinum Caused by Rupture of a Bronchial Artery Aneurysm. *Anales del Sistema Sanitario de Navarra*; 2013.

51. Vosse BA, van Belle AF, de Vries GJ, Das M. Hemomediastinum Due to Spontaneous Rupture of a Mediastinal Bronchial Artery Aneurysm - a Rare Cause of Thoracic Pain. *Respir Med Case Rep* (2014) 12:27-9. Epub 20140314. doi: 10.1016/j.rmcr.2013.12.012.

52. Kim YK, Sung YM, Kim JH, Byun SS, Park YN, Lee SP. Aortic Stent-Graft for a Giant Bronchial Artery Aneurysm with Ultrashort Neck. *Annals of Thoracic and Cardiovascular Surgery* (2014) 20:781-5. doi: 10.5761/atcs.cr.12.02117.

53. Cheng YS, Lu ZW. Bronchial Aneurysm Secondary to Tuberculosis Presenting with Fatal Hemoptysis: A Case Report and Review of the Literature. *J Thorac Dis* (2014) 6(6):E70-2. doi: 10.3978/j.issn.2072-1439.2014.04.07.

54. Nakamura R, Inage Y, Tsuchiya A, Kato T, Yuzawa K, Koizumi M, et al. Bronchial Artery Aneurysm Treated with Video-Assisted Thoracoscopic Surgery. *Annals of Vascular Surgery* (2014) 28(5):1321. e1-. e3.

55. Cao X, He H, Li X, Sun B. Extracorporeal Membrane Oxygenation as a Platform for the Management of Massive Hemoptysis Caused by Bronchial Artery Aneurysm. *Chin Med J (Engl)* (2014) 127(16):3032.

56. Kim JS, Lee SY, Son KH, Kim KW, Choi CH, Lee JI, et al. Bronchial Artery Aneurysm Presenting as Hematemesis and Mediastinal Hemorrhage. *Korean J Thorac Cardiovasc Surg* (2015) 48(4):298-301. Epub 20150805. doi: 10.5090/kjtcs.2015.48.4.298.

57. Rognoni A, Lupi A, Rosso R, Cavallino C, Bongo AS. Giant Bronchial Artery Aneurysm Treated by Coil Embolization and Amplatzer Placement. *Journal of Cardiovascular Medicine* (2015) 16:S66-S7.

58. Samura M, Morikage N, Yamashita O, Murakami M, Suehiro K, Hamano K. Combination of Aortic Stent Grafting and Arterial Embolization for Bronchial Artery Aneurysm Associated with Bronchial-Pulmonary Arterial Fistula. *J Vasc Interv Radiol* (2015) 26(7):1077-9. Epub 2015/06/23. doi: 10.1016/j.jvir.2015.01.035.

59. Shi Y, Hu H, Zhang W, Wang W. Percutaneous Embolization for Ruptured Ectopic Bronchial Artery Aneurysm: A Case Report. *Medicine (Baltimore)* (2015) 94(20):e749. Epub 2015/05/23. doi: 10.1097/md.0000000000000749.

60. Divisi D, de Vico A, Crisci R. Conservative Approach in Bronchial Artery Aneurysm Rupture: A Therapeutic Option. *Archivos de Bronconeumología* (2015) 51(9):473-4. doi: 10.1016/j.arbr.2015.03.012.

61. Kitami A, Sano F, Hayashi S, Suzuki K, Uematsu S, Suzuki T, et al. A Surgical Case of Bronchial Artery Aneurysm Directory Connecting with Pulmonary Artery. *Ann Thorac Cardiovasc Surg* (2015) 21(6):564-6. Epub 20150605. doi: 10.5761/atcs.cr.15-00054.

62. Mahmood RD, Chen ZY, Low TB, Ng KS. A Rare Case of Multiple Bronchial Artery Aneurysms Associated with a Double Aortic Arch. *Singapore Med J* (2015) 56(3):e42-5. doi: 10.11622/smedj.2015050.

63. Di X, Ji DH, Chen Y, Liu CW, Liu B, Yang J. Endovascular Treatment of Ectopic Bronchial Artery Aneurysm with Brachiocephalic Artery Stent Placement and Coil Embolization: A Case Report and Literature Review. *Medicine (Baltimore)* (2016) 95(35):e4461. doi: 10.1097/md.0000000000004461.

64. Izaaryene J, Vidal V, Bartoli JM, Gaubert JY. Multiple Bronchial Artery Aneurysms: Successful Treatment with Ethylene-Vinyl Alcohol Copolymer (Onyx®). *Diagn Interv Imaging* (2016) 97(1):125-7. Epub 20151125. doi: 10.1016/j.diii.2015.05.009.

65. Mohd Alkaf AL, Syed Rasul SH, Abdul Rahman I. Mycotic Bronchial Artery Aneurysmal Rupture in the Early Stage of Lung Abscess: A Case Report. *Med J Malaysia* (2016) 71(2):96-7.

66. Fiorucci B, Simonte G. Treatment of a Bronchial Artery Aneurysm with Placement of an Aortic Thoracic Stent-Graft. *Eur J Vasc Endovasc Surg* (2016) 51(5):717. Epub 20160324. doi: 10.1016/j.ejvs.2016.02.014.

67. Sato K, Fumimoto S, Fukada T, Ochi K, Kataoka T, Ichihashi Y, et al. Bronchial Artery Aneurysm Suggested to Be Caused by Metalic Tracheal Stent Migration. *Surg Case Rep* (2016) 2(1):125. Epub 2016/11/07. doi: 10.1186/s40792-016-0247-1.

68. Song M, Wu H, Jiang J, Wang Ga, Wu S, Ge T. Case Report Mediastinal Bronchial Artery Aneurysm Treated with Aortic Stent and Embolization: Case Report. *Int J Clin Exp Med* (2016) 9(9):18593-600.

69. Wang Z, Xu C, Ding X, Chen J, Xin H. Spontaneous Rupture of a Mediastinal Bronchial Artery Aneurysm Induced by Anticoagulant Agent. *Thorac Cardiovasc Surg Rep* (2016) 5(1):18-20. Epub 20160404. doi: 10.1055/s-0036-1578813.

70. Salamone I, Cavallaro M, Visalli C, Velo M, Barbaro U, Galletta K, et al. Embolization of a Bronchial Artery Aneurysm in a Chronic Obstructive Pulmonary Disease (Copd) Patient with Non-Massive Hemoptysis. *Pol J Radiol* (2017) 82:174-8. Epub 20170331. doi: 10.12659/pjr.899624.

71. Matsumoto T, Uchida T, Ono T, Hirayama K, Fukumura F, Ando H, et al. Bronchial Artery Aneurysm Treated Using Aortic Stent Graft Alone: A Case Report. *Ann Vasc Dis* (2017) 10(2):152-4. Epub 2017/10/17. doi: 10.3400/avd.cr.16-00091.

72. Hsieh CG, Le T, Fogelfeld K, Kamangar N. Bronchial Artery Aneurysm with Associated Bronchial Artery to Pulmonary Artery Fistula: Treatment by Embolization. *J Clin Imaging Sci* (2017) 7:2. Epub 20170127. doi: 10.4103/2156-7514.199052.

73. Ueda R, Kotani Y, Tsujimoto T. A Ruptured Bronchial Artery Aneurysm with Massive Haemoptysis. *Case Reports* (2017) 2017:bcr-2017-220392.

74. Wang J, Zhou G, Liang B, Makamure J, Pan F, Zhao D, et al. Combination of Aortic Stent-Graft and Arterial Embolization for Multiple Bronchial Artery Aneurysms Associated with Diffuse Bronchial-Pulmonary Arterial Fistulas. *J Vasc Interv Radiol* (2018) 29(9):1283-5. doi: 10.1016/j.jvir.2017.12.017.

75. Goh Y, Rajendran PC, Loh SEK, Choong A, Ng SJK, Wee B. Transcatheter Embolization of a Large Mediastinal Bronchial Artery Aneurysm with Short Neck. *ANZ J Surg* (2019) 89(5):597-9. Epub 20171009. doi: 10.1111/ans.14198.

76. Li Y, Gu GC, Liu B, Shao J, Chen Y, Zheng YH. Endovascular Treatment of Multiple Bronchial Artery Aneurysms with Prominent Fistula to Pulmonary Artery in a Patient with Interstitial Lung Disease: A Case Report and Literature Review. *Vasc Endovascular Surg* (2019) 53(6):492-6. Epub 20190424. doi: 10.1177/1538574419845184.

77. Chen Y, Qin W, Zhu Z, Wang X, Yu W, Li F, et al. Interlocking Detachable Coil Embolization for Giant Tandem Bronchial Aneurysms: A Case Report. *Medicine (Baltimore)* (2021) 100(51):e28416. doi: 10.1097/md.0000000000028416.

78. Balar AB, Lakhani DA, Martin D, Smith KT, Kim C. Giant Mediastinal Bronchial Artery Aneurysm Mimicking Mediastinal Mass: A Case Report and Brief Review of the Literature. *Radiol Case Rep* (2022) 17(5):1496-501. Epub 20220303. doi: 10.1016/j.radcr.2022.02.015.

79. Cho Y, Kim JK. A Ruptured Bronchial Artery Aneurysm Presenting with Neurological Symptoms: A Case Report. *The American Journal of Emergency Medicine* (2023). doi: <https://doi.org/10.1016/j.ajem.2023.09.040>.
